# Eosinophilic gastroenteritis in an elderly men associated with antibiotic use post maxillofacial space infection: a case report

**DOI:** 10.3389/fmed.2024.1370674

**Published:** 2024-06-26

**Authors:** Ran Lin, Kangjie Ye, Min Hong, Jiqiang Li, Zhongde Zhang, Xi Zhang

**Affiliations:** ^1^The Second Clinical College, Guangzhou University of Chinese Medicine, Guangzhou, China; ^2^[The Second Clinical College, Guangzhou University of Chinese Medicine, Guangzhou, Guangdong, China

**Keywords:** eosinophilic gastroenteritis, infection, antibiotic, colonic tubulovillous adenoma, eosinophilia

## Abstract

A 79-year-old man underwent operative drainage and 2-week cephalosporin treatment due to a maxillofacial space infection (bilateral submaxillaris, submentum, and left face). However, he experienced anorexia, nausea, vomiting, and emaciation in the following 2 months. It was initially considered that a malignancy might be present, thus a series of examinations were performed. Laboratory investigations showed increases in inflammatory markers and a significant eosinophilia, which seemed to be a hematological system disease. Combined with the gastrointestinal endoscopes and histology examination, the patient was diagnosed with eosinophilic gastroenteritis (EGE). After cessation of antibiotic treatment and administration of corticosteroid, our patient experienced a rapid progress in his clinical condition. Despite the low incidence, EGE should be considered in patients with unknown cause of gastrointestinal disorder, elevated eosinophilia, and so on.

## Introduction

Eosinophilic gastroenteritis (EGE) is an eosinophil-rich gastrointestinal disorder with a low incidence (5.1/100,000) (1), but the causes of eosinophilia have not been identified. In general, eosinophilia typically proves to be reactive, and secondary causes of gastrointestinal eosinophilia are both numerous and more common than primary eosinophilic diseases. Sometimes it may be a paraneoplastic manifestation of malignancies. We report a man with unknown primary eosinophilia and gastrointestinal disorder, who had a clear history of infection and antibiotic use before the onset of his disease. Though some proposed that antibiotic use is an independent risk factor for EGE, related case reports are rare. Here, the reported case showed post-infection antibiotic use may be a disease trigger of EGE. Moreover, a subsequent tissue biopsy revealed tubular villous adenoma of the colon with high-grade dysplasia; but the causal links between them remain unclear and should be further explored.

## Case report

A 79-year-old man was admitted to our hospital with a 20-day history of worsening anorexia, nausea, and vomiting, and lost 15 k in body weight. Two months prior to this episode, the patient presented to the emergency department with maxillofacial swelling and pain. A face and neck CT scan showed features of inflammatory diseases in bilateral submaxillaris, submentum, and left face. Blood counts during hospitalizations revealed an elevated white blood cell count of 11.42 × 10^9^ per L (normal 3.50–9.50) and a slightly lower eosinophil count of 0.00 × 10^9^ per L (normal 0.02–0.52). After undergoing operative drainage and 2-week cephalosporin treatment based on the drug-sensitivity result of pus, he was completely recovery.

Two months after discharged, the patient gradually experienced anorexia, nausea, vomiting, and a fast loss of weight. When visiting our hospital, he was confined to a wheelchair, experiencing urinary incontinence, constipation with dry stool, and no fever or night sweats. The patient had a history of being cured of tuberculosis, had no recent travel or adverse reactions to any allergens. During the physical examination, the patient’s abdomen appeared soft and flat with no mass, tenderness, or rebound tenderness. Laboratory investigations showed leukocytosis (40.48 × 10^9^ per L; normal 3.50–9.50), accompanied by significant eosinophilia at 30.24 × 10^9^ per L (normal 0.02–0.52), a sharp increase in IgE concentration (1395.00 IU/mL; normal <100), a decrease in C-reactive protein concentration (61.40 mg/L; normal 0–6), and hemoglobin concentration (81.00 g/L; normal 130–175), and positive hydatid IgG antibody and cysticercus IgG antibody. The results of the fecal and gastric juice occult blood tests were positive. CA19-9, CEA, and AFP concentrations were normal.

**Figure 1:**
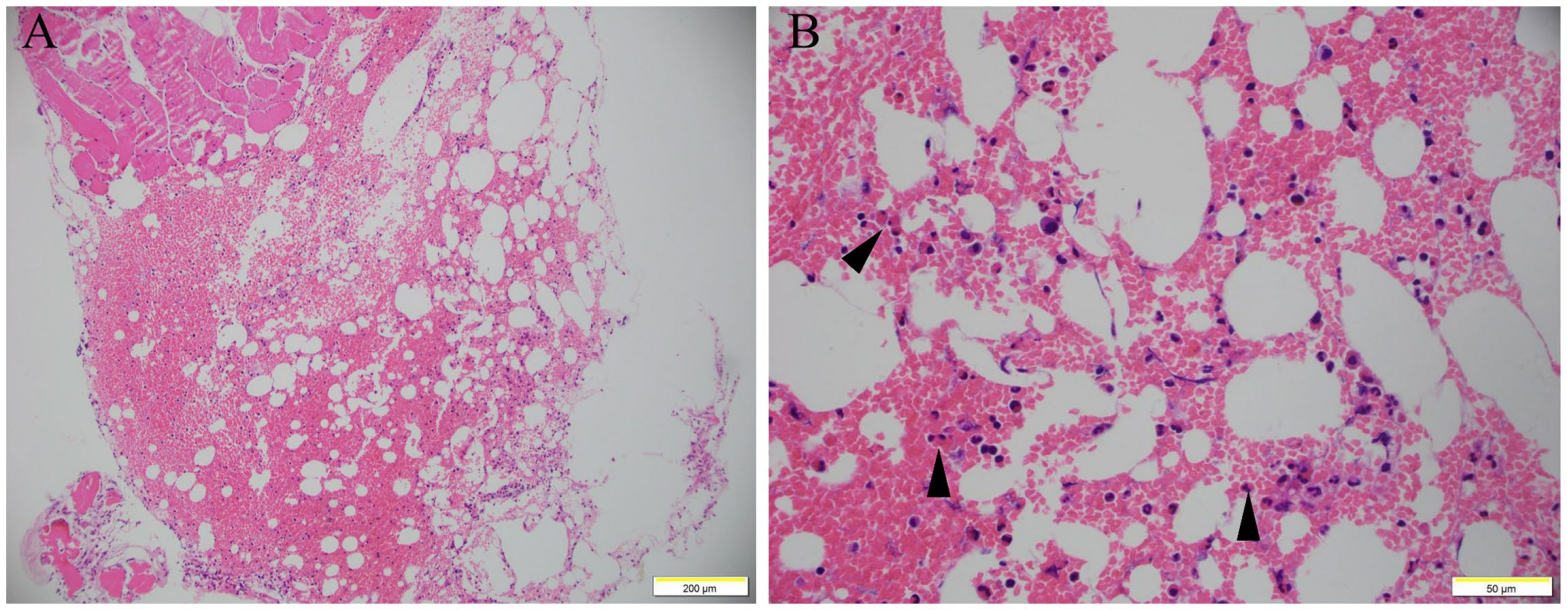
Bone marrow smear. Bone marrow smear showed increased red blood cells, scattered eosinophils infiltration (arrow), and MF grade 2 of reticulum fiber staining (**A**, H&E staining, 100×; **B**, H&E staining, 400×).

Bone marrow biopsy showed increased red blood cells, scattered eosinophils infiltration, and MF grade 2 of reticulum fiber staining (Figure 1), which ruled out lymphocytic and myeloproliferative forms of hypereosinophilic syndrome. BCR/ABL1 fusion gene testing of the patient’s peripheral blood was negative. Screening tests for autoimmune diseases did not reveal any abnormalities. The CT scan and 3D reconstruction showed cholecystolithiasis with chronic cholecystitis, old pulmonary tuberculosis, nodular goiter, hepatic cyst, adrenal hyperplasia, simple renal cyst, and bladder calculi. Repeated parasitological examinations of different stool samples showed no positive results.

**Figure 2:**
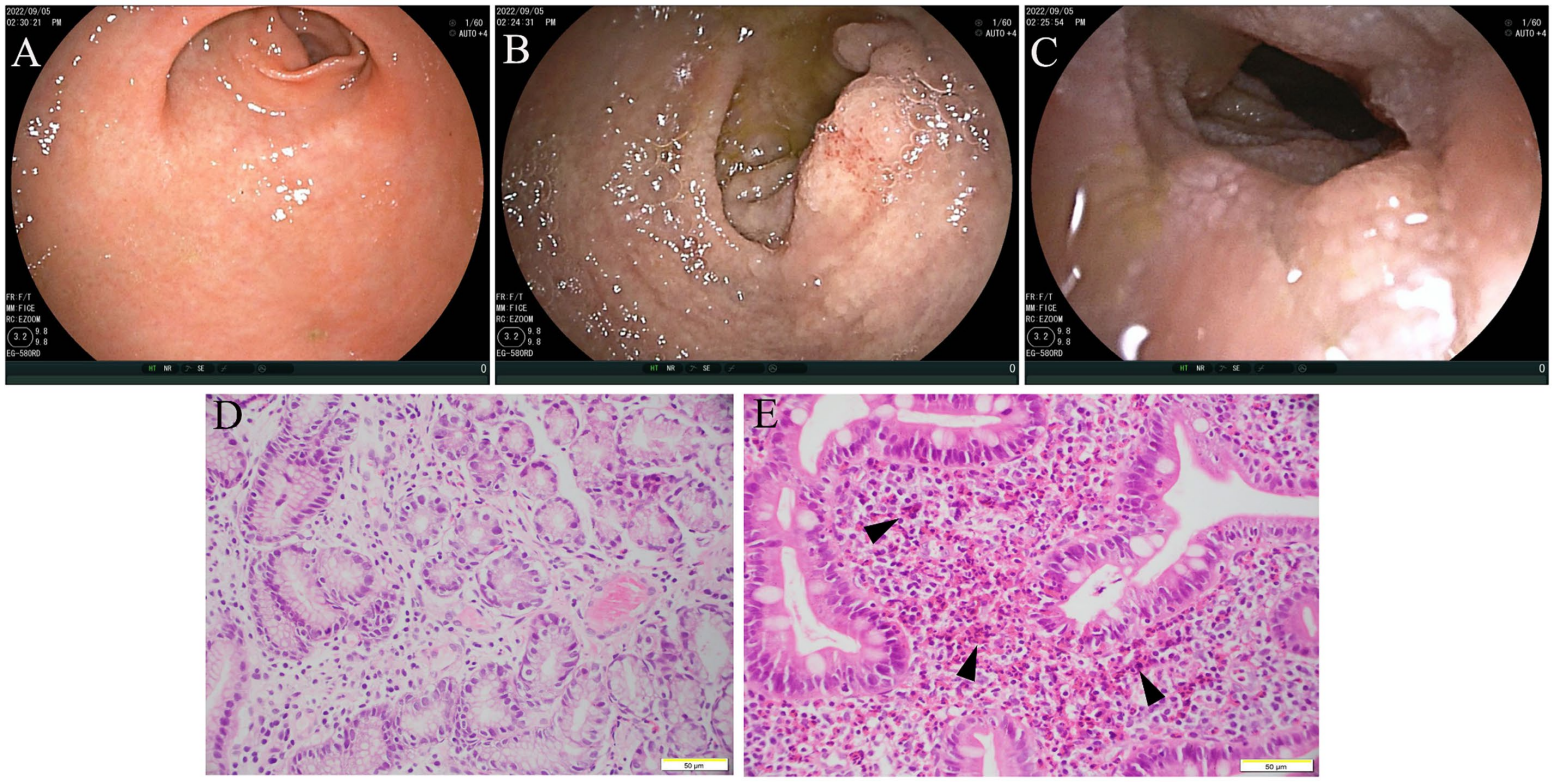
Gastroscope and histology examination (2022/09/05). The gastroscope views showed erythema in the gastric antral mucosa **(A)**, hyperemia and edema in the mucosa of gastroduodenal junction **(B)**, and edema in the descending part of duodenum **(C)**. Histology of the gastric antrum mucosa revealed chronic mucositis **(D)**. Histology of the descending part of duodenum showed the villi structure of descending duodenum is near normal. Numerous eosinophils are evenly dispersed in the interstitium (>200 eos/hpf) (arrow); inflammatory infiltrates also contain macrophages, plasma cells, and scattered neutrophils **(E)**.

**Figure 3:**
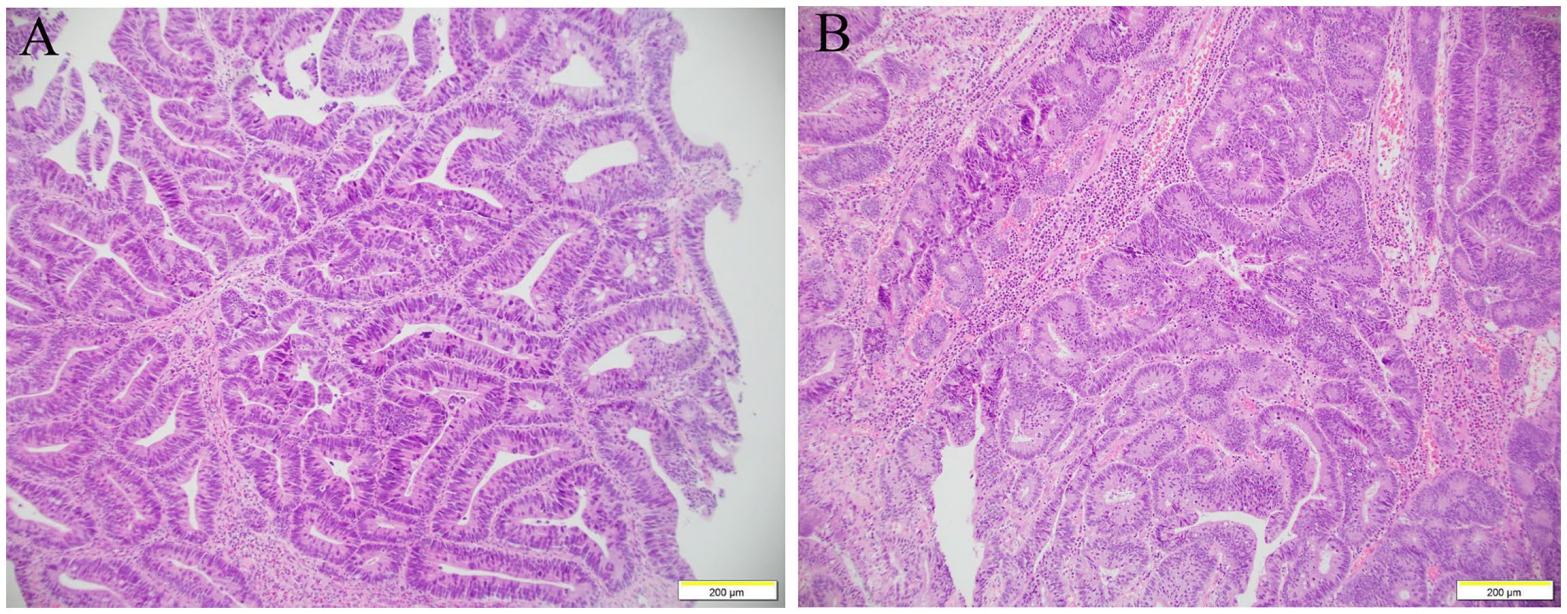
Histology examination of colon. The glandular structure of ascending colon was irregular, and the glandular epithelial cells were increased in layers and arranged disorderly, with severe hyperplasia of the nucleus and pathological nuclear fission, increased eosinophils are dispersed in the interstitium **(A)**. The glands were crowded arrangement and confluent, some of them showed cribriform structure, increased cell layers, and moderate to severe nuclear dysplasia **(B)**.

The diagnosis of gastrointestinal endoscopy demonstrated esophageal ulcer A1, chronic non-atrophic gastritis, and duodenitis. Histopathological analysis of intestinal mucosa biopsy specimens showed marked eosinophilic infiltration of the descending duodenum mucosa with more than 200 eosinophils per high-power field (Figure 2). Enteroscopy revealed a 6 mm × 4 mm flat poly with type 1 NICE features in the caecum; a 15 mm × 15 mm polypoid mass in the ascending colon with a less smooth surface, sunken center, and NICE type 2–3; a 60 mm × 50 mm polypoid mass around the hepatic flexure of transverse colon with smooth surface and NICE type 2; a 15 mm × 10 mm polypoid mass with type 2 NICE features in the sigmoid colon. Histopathological analysis of these polyps revealed tubular adenomas with pathological epithelial hyperplasia ranging from mild to severe, and increased eosinophils were dispersed in the interstitium (Figure 3). Based on the combination of biological, radiological, and histological evidences, the patient was diagnosed with EGE.

The patient was started on methylprednisolone (16 mg/day) with rapid symptom improvement and a significant decrease in serum eosinophilia level. After about 20 days of treatment, the patient was discharged from the hospital with a recommendation to undergo a selective operation to remove intestinal polyps. The dose of oral prednisolone was reduced by 4 mg/day every week.

**Figure 4:**
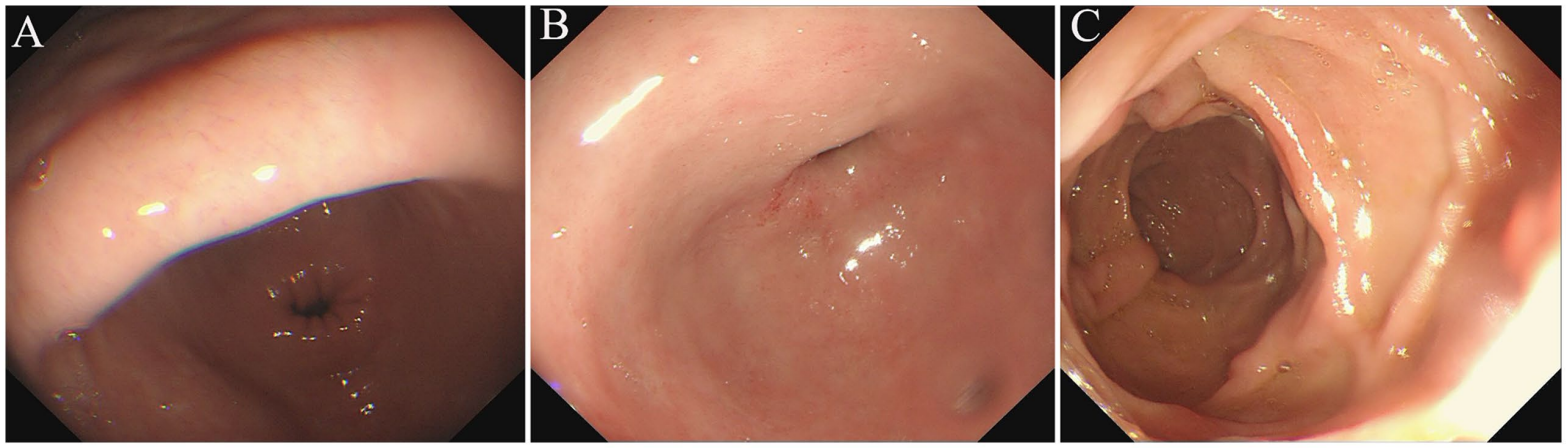
Repeated Gastroscope examination (2022/12/09). The gastroscope views showed hyperemia in the gastric antral mucosa **(A)**, no abnormality in the mucosa of duodenal bulb and upper part of pars descendent **(B,C)**.

Two months later, he presented to our hospital for treatment with abdominal distension rather than nausea or vomiting. A complete blood picture showed a slight increase in the total count of white blood cells (9.78 × 10^9^ per L) and eosinophilia of 1.94 × 10^9^ per L (19.8%). The repeated gastroscope revealed chronic non-atrophic gastritis, mucosal repair improvement of the bulb, and descending duodenum (Figure 4). The enteroscopy appearance of polypoid masses in the ascending and transverse colon were the same as before. Since there was an absolute operative indication, our patient was treated with endoscopic submucosal dissection (ESD) and endoscopic mucosal resection (EMR). Postoperative pathological results of the transverse colon polyp indicated the tubulovillous adenoma with high-grade dysplasia of focal epithelium. Immunohistochemical staining showed a positive reaction of Desmin in smooth muscle with the P53 and the Ki67 indexes (approximately 70%). After treatment with anti-allergic and nutrition support, the patient’s eosinophil count returned to normal, and his clinical condition gradually improved with 5-k weight gain.

## Discussion

Eosinophils are normal constituents of the gastrointestinal tract that are present throughout the distal part of the squamous esophagus (2). Under normal physiological conditions, eosinophils participate in regulating gastrointestinal tract microbiome and contribute to tissue homeostasis, such as maintenance of epithelial barrier function, as well as contributing to defense against pathogenic parasites and bacteria (2, 3). In eosinophilic gastrointestinal disorders, pathogens or allergens cause excessive eosinophils recruitment, leading to epithelial damage (4). EGE is one of the rare distal eosinophilic gastrointestinal diseases (EGIDs), which sometimes occurs as a comorbidity in patients with eosinophilic esophagitis (5, 6). Because of the low prevalence of distal EGIDs and limited understanding of pathophysiology, to date, there are no uniform diagnostic criteria or standardized guidelines for monitoring, management, and treatments.

Eosinophilic gastroenteritis is characterized by eosinophilia and eosinophil infiltration into alimentary tract mucosa, as well as various digestive symptoms. The diagnosis of EGE is based on the clinical symptoms and histological findings, excluding identified secondary causes of eosinophilia, such as parasitic infection, drug reactions, parasitic infections, and malignancy. The clinical presentation, course, and prognosis of EGE depend on the location and depth of eosinophilic involvement in the different layers of the gastrointestinal tract (7). Patients with EGE suffer from impaired quality of life and face barriers to treatment, including delayed diagnosis, misdiagnosis, and few therapeutic options (8, 9). There are three patterns of eosinophilic gastroenteritis: mucosal, mural, and serosal. Patients with mucosal involvement suffered vomiting, blood in the stool, iron deficiency anemia, malabsorption, and protein-losing enteropathy, as happened in our patient. Patients with muscle layer or serosal involvement typically present with obstruction or ascites. The pathogenesis of EGE is not fully understood, but food allergies and intestinal dysbiosis have been implicated. Epidemiologic and clinical features suggest that EGE is associated with allergic diseases (10). Although the serum IgE level was distinctly elevated to 1,395 IU/mL (upper limit of normal, 173 IU/mL) at the time when diagnosed with EGE, our patient did not have any history of allergy or hypereosinophilia. Since infection is usually associated with blood and tissue eosinophilia, stool examination was conducted to exclude parasitic infestation.

So far, the number of eosinophils essential for a diagnosis is not well defined and may depend on the mucosal distribution of the biopsy (11, 12). A total of 30 eosinophils per high-power field was considered as a referential number to make a diagnosis of eosinophilic gastritis. Therefore, histologic examination showed roughly normal villus structure and eosinophilic infiltration (>200 per HPF) into the duodenum mucosa, compatible with mucosal EGE. It is important to note that high blood eosinophil counts at diagnosis were associated with a high risk of clinical relapse (13), but nearly half of the EGE patients had no increase in peripheral eosinophils (14). Thus, peripheral eosinophilia’s absence is insufficient to rule out EGE. Moreover, patients with extensive disease may be more susceptible to relapse, whereas those with only lower gastrointestinal tract affection have a better prognosis (15).

The goal of therapy is to reduce the absolute eosinophil count and mitigate tissue infiltration and eosinophil-mediated tissue damage (15). Dietary therapy is recommended as a first-line treatment for cases of EGE associated with food allergy (16). If dietary therapy fails to achieve an adequate clinical response or non-drug food allergy, corticosteroids can be used as first-line drug therapy (17). Corticosteroids are the primary treatment for most EGE patients due to their effects on improving symptoms by rapidly reducing in the eosinophil counts. Because EGE often recurs after corticosteroid discontinuation, an ongoing maintenance treatment might be required to prevent from symptoms relapsing during or after drug tapering. Since our patient had significant eosinophilia on his first admission, a slight recurrence of blood eosinophilia in 2 months seemed to be predictable.

There are no established causes recognized for EGIDs. However, some risk factors may include hypersensitivity reaction to a certain food, medications (such as vancomycin, allopurinol, lamotrigine, carbamazepine, and trimethoprim-sulfamethoxazole) (18), and a family or personal history of preexisting allergic diseases (such as asthma, allergic rhinitis, or eczema) (19, 20).

Eosinophilic gastroenteritis associated with antibiotic use has rarely been reported. A retrospective study of 23 patients with EGE showed 38.4% had used antibiotics within 1 month before diagnosis, and then logistics multivariate analysis showed that antibiotic usage was an independent risk factor for EGE (21). There are no reports showing EGE occurring secondary to cephalosporin antibiotic administration, although antibiotic is associated with gastrointestinal side effects and anaphylaxis. In this case, the patient was diagnosed at an advanced age, without a history of allergic diseases.

In fact, although uncommon, cytoplasmic eosinophilia may be encountered in conventional villous/tubulovillous (22). Moreover, as shown in this case, histologic examination revealed colonic tubulovillous adenoma with high-grade dysplasia, which is preneoplastic lesion that leads to colorectal cancer. Notably, solid tumors can lead to eosinophilia as well (23), mainly in lung cancer, digestive system tumors, neck tumors, kidney tumors, and so on. Eosinophil is an integral part of the immune landscape of various tumors, especially mucosal tumors (24), where they can migrate to tumor sites in the tumor microenvironment with or without eotaxins (25). The degree of infiltration varies and may be limited to the area of the tumor or emanate away from it. Tumor infiltrated eosinophils can influence the tumor microenvironment resulting in either anti-tumorigenic or pro-tumorigenic effects (26). The presence of eosinophils within the tumor microenvironment still be of unclear prognostic significance (23). In terms of management of tumor-associated eosinophilia, the primary neoplasms should be treated seriously, whose resection followed by radiotherapy and chemotherapy can improve eosinophilia and infiltration. It is conceivable that because the eosinophilia regress upon oral prednisolone rather than the treatment of gross total excision of the tumor. In addition, EGE cases related to precancerous lesions are particularly rare, and therefore the relevance of EGE to tumors has not been explored. More evidence is needed to confirm whether EGE is an early clinical manifestation of partial gastrointestinal tumors.

## Conclusion

Eosinophilic gastroenteritis is an uncommon and heterogeneous gastrointestinal disease that has a complex pathogenesis and is often under-diagnosed. In recent years, the prevalence of EGE has increased gradually. The exact causes of EGE are not fully understood. There was a clear history of recent antibiotic usage for local infection in our patient, hence, the temporal order between medication and EGE leads to the speculate that antibiotic use may be one important inducible factor of EGE. More evidences are needed to investigate the underlying mechanism.

## Data availability statement

The original contributions presented in the study are included in the article/supplementary material, further inquiries can be directed to the corresponding author.

## Ethics statement

The studies involving humans were approved by the Second Affiliated Hospital of Guangzhou University of Chinese Medicine. The studies were conducted in accordance with the local legislation and institutional requirements. The participants provided their written informed consent to participate in this study. Written informed consent was obtained from the individual(s) for the publication of any potentially identifiable images or data included in this article.

## Author contributions

RL: Writing – original draft, Writing – review & editing. KY: Writing – original draft, Writing – review & editing. MH: Writing – review & editing, Data curation, Formal analysis, Investigation. JL: Conceptualization, Methodology, Supervision, Writing – review & editing. ZZ: Conceptualization, Methodology, Supervision, Writing – review & editing. XZ: Writing – original draft, Writing – review & editing.
